# Flexi-pharma: a molecule-ranking strategy for virtual screening using pharmacophores from ligand-free conformational ensembles

**DOI:** 10.1007/s10822-020-00329-7

**Published:** 2020-07-12

**Authors:** Isaias Lans, Karen Palacio-Rodríguez, Claudio N. Cavasotto, Pilar Cossio

**Affiliations:** 1 Biophysics of Tropical Diseases Max Planck Tandem Group, University of Antioquia UdeA, Calle 70 No. 52-21, Medellín, Colombia; 2 Computational Drug Design and Biomedical Informatics Laboratory, Translational Medicine Research Institute (IIMT), CONICET-Universidad Austral, Pilar, Buenos Aires Argentina; 3 Facultad de Ciencias Biomédicas, and Facultad de Ingeniería, Universidad Austral, Pilar, Buenos Aires Argentina; 4 Austral Institute for Applied Artificial Intelligence, Universidad Austral, Pilar, Buenos Aires Argentina; 5 Department of Theoretical Biophysics, Max Planck Institute of Biophysics, 60438 Frankfurt am Main, Germany

**Keywords:** Ligand-free pharmacophore, Virtual screening, Dynamics, Drug discovery, Enrichment, Affinity map

## Abstract

Computer-aided strategies are useful for reducing the costs and increasing the success-rate in drug discovery. Among these strategies, methods based on pharmacophores (an ensemble of electronic and steric features representing the target active site) are efficient to implement over large compound libraries. However, traditional pharmacophore-based methods require knowledge of active compounds or ligand–receptor structures, and only few ones account for target flexibility. Here, we developed a pharmacophore-based virtual screening protocol, Flexi-pharma, that overcomes these limitations. The protocol uses molecular dynamics (MD) simulations to explore receptor flexibility, and performs a pharmacophore-based virtual screening over a set of MD conformations without requiring prior knowledge about known ligands or ligand–receptor structures for building the pharmacophores. The results from the different receptor conformations are combined using a “voting” approach, where a vote is given to each molecule that matches at least one pharmacophore from each MD conformation. Contrarily to other approaches that reduce the pharmacophore ensemble to some representative models and score according to the matching models or molecule conformers, the Flexi-pharma approach takes directly into account the receptor flexibility by scoring in regards to the receptor conformations. We tested the method over twenty systems, finding an enrichment of the dataset for 19 of them. Flexi-pharma is computationally efficient allowing for the screening of thousands of compounds in minutes on a single CPU core. Moreover, the ranking of molecules by vote is a general strategy that can be applied with any pharmacophore-filtering program.

**Electronic supplementary material:**

The online version of this article (10.1007/s10822-020-00329-7) contains supplementary material, which is available to authorized users.

## Introduction

During the past two decades, the implementation of computational tools in drug discovery has increased. Many pharmaceutical companies have included computational methods in their drug discovery pipelines [1, 2]. These methods have been useful to decrease the costs of drug discovery by reducing the number of compounds to test in experimental assays [3]. Their implementation in drug-screening protocols increases the rate of success of finding active compounds and reduces the false negatives from high-throughput compound screening [1, 4–10].

Among the computational strategies for virtual screening the most popular are the quantitative structure-activity relationship (QSAR) [11, 12], docking [1, 8, 13, 14], and pharmacophore-based strategies [15–19]. QSAR defines a quantitative relationship (mathematical equation) between the electronic-structural characteristics and the activity from a set of known active compounds. The resultant relationship is employed to identify new active compounds [11, 12]. However, the QSAR prediction will only work over compounds that are physico-chemically similar to those in the training set [11]. Alternatively, molecular docking is widely used in virtual screening for predicting the conformations of ligand–receptor complexes and binding scores [1, 8, 20]. The search of the ligand–receptor conformations requires a significant amount of computational resources. Nonetheless, with multi-node computing clusters high-throughput docking simulations are commonly used.

Pharmacophore-based strategies require less computational resources than molecular docking, making them more suitable for exploring large compound libraries [15, 16, 21]. These methods search for molecules that match an ensemble of electronic and steric features (pharmacophore) required to be a ligand of a specific biological target. There are two standard types of methods to define a pharmacophore (i) ligand based or (ii) structure based. The ligand-based methods use an alignment of known active compounds to identify common features for building the pharmacophore [22]. Similarly to QSAR, this strategy has the disadvantage of requiring known active compounds and the filtered molecules will cover only the pharmacophoric space associated with the initial training set. On the other hand, instead of requiring known active compounds, structure-based strategies require protein structures to be available. Some strategies use ligand–receptor structures to define pharmacophores using the most relevant interactions present in the complexes [23–28].

Other methods introduce the flexibility of the receptor using molecular dynamics (MD) simulations of the ligand–receptor complex [19, 28, 29] and define representative pharmacophores from the MD conformations. Wieder et al. [19] define the representative pharmacophores by grouping types and number of features but ignoring their spatial arrangement. They use a common hits approach (CHA), scoring the molecules according to the number of representative pharmacophores that match at least one molecule conformation (conformer). Whereas Polishchuck et al. [29] present a conformers coverage approach (CCA), scoring the molecules according to the number of its conformers that match any representative pharmacophore. However, we note that in these cases the pharmacophores are defined from the ligand–receptor interactions, and hence, the pharmacophoric space remains limited to the interactions observed in the complexes.

There are several ligand-free methods that define pharmacophores from the receptor structure (without ligand). Mortier et al. designed a strategy to identify relevant interactions in protein interfaces using crystallographic structures [15]. Another set of methods creates a consensus pharmacophore model from several conformations from MD simulations of the ligand-free receptor [18, 30, 31]. However, the performance of these methods, in terms of the enrichment of several benchmark systems, has not been evaluated. Considering all this information, most pharmacophore-based methods have some of these limitations: (i) they can produce biased results (due to the training sets or the ligand–receptor structures), (ii) some methods produce only a binary output: a list of compounds that match the pharmacophores, but without giving each molecule a score or rank, and, (iii) few methods include the flexibility of the receptor in the pharmacophore determination, leaving room for their improvement.

In this study, we developed a pharmacophore-based methodology (Flexi-pharma) for virtual screening that overcomes the aforementioned limitations. Inspired by conformational-prediction tools that take into account the receptor flexibility [32, 33], we use multiple conformations from MD simulations. We develop a novel protocol to generate pharmacophores from the ligand-free receptor conformations, and screen compounds. A molecule is awarded a vote for each receptor conformation that has one or more pharmacophores that are matched by the molecule. In the end, the evaluation of the MD conformations results in a number of votes for each molecule that is used as a score. We first optimized the protocol using four systems, then we tested the methodology over sixteen additional systems. We find that the Flexi-pharma list of votes can on average discriminate active compounds from decoys, resulting in an enrichment of the dataset.

## Methods

### Flexi-pharma protocol

**Fig. 1:**
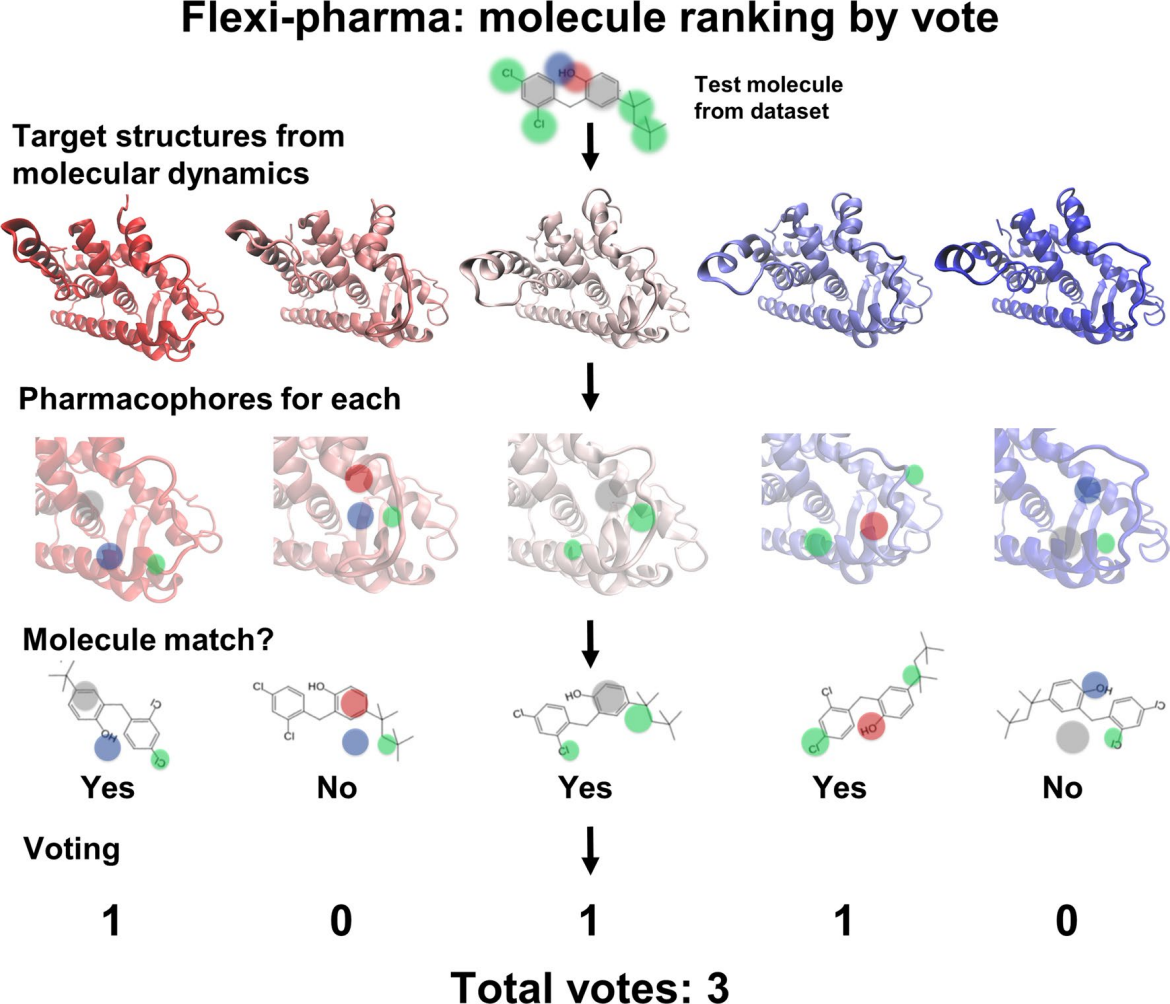
Schematic representation of the Flexi-pharma protocol for one test molecule. First, an MD simulation is performed. Then, a set of pharmacophores is determined for each MD conformation of the receptor. Then, Pharmer [34] is used to identify if the molecule matches any pharmacophore from each MD conformation; if so, a vote is given to the molecule. Finally, we sum the votes from the set of MD conformations. The green, blue, red and gray circles are hydrophobic, H-bond donor, H-bond acceptor and aromatic features, respectively

The Flexi-pharma protocol consists on the following steps: (1) run an MD simulation starting from a crystal structure; (2) select a set of MD conformations of the ligand-free receptor; (3) build a set of pharmacophores for each conformation; (4) use Pharmer [34] to screen a compound library finding the molecules that match any pharmacophore; (5) give a ‘vote’ to the molecules that match at least one pharmacophore for each conformation and (6) add the votes from the MD conformations. The total number of votes per molecule will be used as a score to rank the compounds from the library (i.e., the molecules with more votes are considered more active). A schematic summary of the protocol is shown in Fig. 1.

In the following, we will explain in detail the steps to generate the pharmacophore set and screen the compounds.

#### Pharmacophore screening

##### Affinity maps

To generate the pharmacophore set, we start from a 3D conformation of the receptor (extracted from the MD simulation). We use autogrid4 [35] to calculate affinity maps (a grid map which allows to identify the most favorable positions of atom-types in the binding pocket) around the active-site. We use several atom-types: hydrogen bond (H-bond) donor, H-bond acceptor, hydrophobic, aromatic and charged atoms. Because autogrid4 does not calculate a unique H-bond acceptor affinity map, the features involving this atoms type are obtained by combining the affinity maps for O, S and N H-bond acceptors. Each affinity map is calculated over a box centered at a point inside the binding-site. The box centers (i.e., active space centers) for each benchmark system (see “Methods” section) are indicate in the Table S1. The box size for the benchmark systems is 12.5 × 12.5 × 12.5 Å using grid cells of 0.25 Å.

##### Receptor specificity

We found that some affinity maps should be discarded from the pharmacophore determination when their grids show a flat landscape (i.e., the distribution of the affinity-energies is very broad). A flat affinity landscape indicates a low specificity of the active site towards the corresponding atom type. To estimate the flatness of the affinity-energy landscape, we calculate the histograms of the negative affinity-energy for each grid map using a bin size of 0.01 kcal/mol (units from autogrid4). Histograms with heavy tails, measured by the kurtosis, are associated to flat affinity-landscapes. The value of the kurtosis for discarding an affinity map depends on the atom type. H-bond acceptor, aromatic and hydrophobic affinity maps having histograms with kurtosis higher than 3 are no longer considered in the pharmacophore determination. For the H-bond donor and charged atom types, a different kurtosis criterion was used. Our results indicate that the majority of affinity maps of H-bond donors show kurtosis larger than 3, a possible explanation for this could be because of the large number of cells with high affinity for the H-bond donor group. Therefore, to discard the H-bond donors affinity maps a higher kurtosis of 4.5 was used.

For charged atom types, we used the affinity map to describe two interactions: ion-ion or ion-dipole. Therefore, each affinity map of a charged atom type is considered twice with different selection criteria. For the ion-dipole interaction, we discarded affinity maps with kurtosis greater than 23. For the ion-ion interaction, we discarded affinity maps with kurtosis greater than 50. We note that the pharmacophore features from the ion-ion interaction are defined differently than all the rest atom types (see below).

**Fig. 2:**
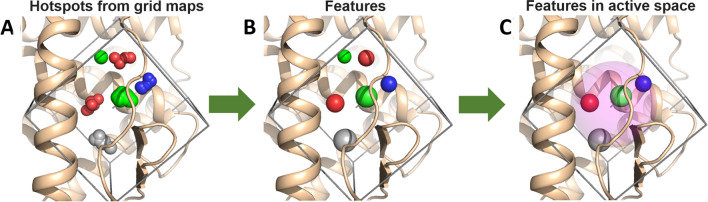
Pharmacophore building. Pharmacophores are obtained from the binding-site hotspots. **a** Hotspots are identified by clustering the grids with highest *x*% percentage of affinity-energy from the affinity maps (enclosed in the box). e.g., for 5 atom types (H-bond acceptor (red), H-bond donors (blue), hydrophobic (green), aromatic (gray) and charge atoms (not shown for sake of simplicity)) are shown. **b** Then, the center of mass (energy-weighted) and the radius of gyration, for each hotspot, are calculated, and used to define the pharmacophoric features (atom type, center of mass and the radius of gyration). **c** Finally, the set of pharmacophores is obtained from all possible combinations of 3 features which centers of mass are located inside of a predefined sphere (pink) of radius 5 Å (active space) that is centered at the grid-map center. In this study, three active spaces were used in each binding site. Therefore, the process (involving the steps A, B and C) was repeated for three sets of grid affinity maps with different centers

##### Hotspots from a grid-percentage threshold

After the receptor specificity is taken into account by discarding the flat affinity grids, we define a grid-percentage threshold *x*% to determine the hotspots for each atom type. The percentage threshold used to determine the hotspots is an input parameter (*x*%). This parameter is a percent of total number of grid cells with negative affinity-energy. Therefore, the best *x*% cells with negative energy are selected. We named these selected grid cells: hotspots (e.g., Fig. 2a).

##### Feature determination

After selecting *x*% of the cells using the grid-percentage threshold, we cluster the cells to generate the pharmacophoric features. A feature is defined by a center, a radius of gyration, an atom type and in some cases a direction. We cluster the grids from each hotspot by grouping all cells that are adjacent to each other in space and belong to the best *x*% of affinity-energy. For each cluster, the center of mass weighted by the affinity-energy and the radius of gyration are calculated (Fig. 2b). In addition, due to the chemical nature of the H-bonds, which relates certain atoms at a specific angle, for H-bond donors and H-bond acceptors, a direction-vector is calculated for each cluster. This vector is defined between the center of mass of the cluster and the closest atom-type counterpart belonging to the receptor.

The above feature-definition is done for all atom types apart for the description of the ion-ion interactions. For this case, an electrostatic feature was assigned over atoms CZ, NZ, CD and CG atoms of Arg, Lys, Glu, Asp, respectively, with a radius of gyration of 4.0 Å. Additionally, if the centers of mass of two features from different atom types are closer than 1 Å, then the two features are not allowed to coexist in the same pharmacophore.

##### The active space

The affinity maps can involve regions outside the binding site (physically separated from the binding pocket or excessively exposed to the solvent). Therefore, taking all the features directly from the grid affinity maps could increase the probability of creating irrelevant features/pharmacophores. To overcome this issue, we define an active space using a sphere, and its center defines the grid-map center (i.e., the active space sphere’s center is the same as the grid-map center). All the features which are centered outside of the sphere are not considered for the pharmacophores definition (transparent pink circle in Fig. 2c). The active space radius depends on the size and shape of the binding pocket. A larger radius increases the probability of finding false positives in the virtual screening. Thus, we use a small radius of 5 Å for all systems. Furthermore, we use several active spaces to cover all the relevant regions of the active site and avoid regions beyond the binding site. Having several active spaces implies that the grid-map is calculated over different regions of the binding site. For the benchmarks used in this study, three active spaces were enough to cover each binding site. The center of each active space is presented in the Table S1. Therefore, the process showed in the Fig. 2 (steps from A to C) was carried out for three different active spaces. We clarify that we have defined the active space centers using previous knowledge of the system’s binding site, such as previous mutational analysis of the binding site or ligand–receptor interactions from crystallographic structures.

##### Volume exclusion

Molecules matching the pharmacophoric features can present steric clashes with the receptor. For this reason, in the pharmacophore determination, we have included a volume-exclusion criterion. For each backbone heavy atom and side chain center of mass from the receptor, we define a feature of volume exclusion with radius of gyration of 0.5 and 1.5 Å, respectively. The radii are small to allow for slightly larger ligand groups to fit inside the binding site. These volume-exclusion features are included in all pharmacophores used for the molecule screening.

##### Pharmacophores

A pharmacophore is obtained by combining three features that are calculated from the different affinity maps and active space, together with the volume exclusion features. Therefore, we create a set of pharmacophores by generating all possible combinations of three features, resulting in multiple pharmacophores for each receptor conformation.

##### Molecule screening

Once we have generated the set of pharmacophores for each receptor conformation, we use the Pharmer program [34] to search the compounds that match at least a pharmacophores of the set. Pharmer is a computational tool for the pharmacophore search inspired in object recognition methods from computer vision. The principal attribute of Pharmer is its efficiency, being able to screen databases of millions of molecules in a few seconds [34].

#### Ranking by vote of the receptor conformation

In the Flexi-pharma protocol, a molecule is awarded a vote for each receptor conformation that has one or more pharmacophores that are matched by the molecule. Here, we consider tautomers (or different protonation states) as the same molecule. The method has been inspired by consensus docking strategies that assign votes to the outcome from different docking programs [36]. All conformations are evaluated and a list of votes allows us to rank the molecules according to the number of times a molecule matches a pharmacophore from a different conformation. Details of the MD simulations used to sample the conformational space of the receptor are provided in the Methods.

### Benchmark systems

We use two sets of benchmark systems: (i) a training set to study and optimize the protocol parameters and, (ii) a test set to evaluate the performance of Flexi-pharma. For each system, one or two crystal starting structures were used (see details below). In addition, a compound library was selected for each system with ligands (active compounds) and decoys (molecules with physicochemical similarity to the ligands but structurally different). We note that the libraries contain tautomers, or different protonation states, of the ligands or decoys. In this study, the tautomers of a molecule were considered as different conformations of the same molecule. Therefore, in our voting scheme, a molecule is assigned a vote when at least one tautomer (or protonation state) matches a pharmacophore from an MD conformation. Due to this consideration, the number of ligands and decoys reported below could be lower than those reported in the original molecule database.

#### Training set

The Flexi-pharma protocol was applied over four diverse benchmark systems: cyclin dependent kinase 2 (CDK2), estrogen receptor α (ER), cyclooxygenase-1 (COX) and glycinamide ribonucleotide transformylase (GAR). The training systems were chosen in terms of the availability of complete 3D structures of the receptors. By using the complete receptor structure, we eliminate possible errors coming from the modeling of gaps present in the crystal structures. For each system, two crystal structures with conformational differences in the active site were chosen.

##### *Cyclin dependent kinase 2 (CDK2)*

Two structures from protein data bank (PDB) were used, 1FVV [37] and 4KD1 [38]. For this system, the Directory of Useful Decoys (DUD) [39] was used. The ligand–decoy compound library contains 50 unique ligands and 1779 decoys.

##### *Estrogen receptor* α *(ER)*

The structures with PDB code 1XP9 [40] and 3ERT [41] were selected. Because these structures are bound to antagonists, a ligand–decoy compound library for antagonists from NRLiSt BDB [42] was used. The dataset contains 133 ligands and 6555 decoys.

##### *Cyclooxygenase−1 (COX)*

Two structures with PDB code 2OYU [43] and 3KK6 [44] were selected. A ligand–decoy compound library from the database of useful decoys: Enhanced (DUD-E) [45] was used. The dataset contains 115 ligands and 7118 decoys.

##### *Glycinamide ribonucleotide transformylase (GAR)*

The 1NJS [46] and 1RC0 structures from the PDB were selected. The dataset was taken from DUD-E [45]. The compound library contains 50 ligands and 2694 decoys.

#### Test set

To evaluate the performance of the Flexi-Pharma protocol, we applied the method over 16 additional systems (for a total of 20 systems, including the training set). The test systems are shown in Table 1 together with the PDB structure, and number of ligand/decoys in the dataset. Structures of the targets NRAM, HSP90a, HXK4, FA7, KITH, FABP4 and PA2GA were prepared as in ref. [47]. For the remaining systems, the targets were downloaded from the PDB and missing atoms were added with the PyMOL mutagenesis tool [48]. The protocol for the protonation of these targets is described in the “Molecular dynamics” section. The active space centers for each system are shown in Table S1. For all the systems in the test set, ligand–decoy compound libraries were taken from DUD-E [45] (see columns 3 and 4 in Table 1). The protonation state of all molecules was kept as in the original dataset. Ligands in DUD-E libraries cover a wide range of binding affinities ($K_i$ from 0.12 pM to 4.9 mM and IC50 from 0.55 pM to 997 nM [45]), which allows for the protocol to be tested on both strong and weak binders.

**Table 1:** Ligand/decoy datasets for targets in the test set

Target name	PDB ID	Ligands	Decoys
Neuraminidase (NRAM)	1B9V	98	6199
Heat Shock Protein 90-alpha (HSP90a)	1UYG	88	4848
Hexokinase Type IV (HXK4)	3F9M	92	4696
Coagulation Factor VII (FA7)	1W7X	114	6245
Thymidine kinase (KITH)	2B8T	57	2850
Fatty Acid Binding Protein Adipocyte (FABP4 )	2NNQ	47	2749
Phospholipase A2 (PA2GA)	1KVO	99	5146
Beta-lactamase (AMPC)	1L2S	48	2832
FK506-binding protein 1A (FKB1A)	1J4H	111	5800
Leukocyte adhesion glycoprotein LFA-1 alpha (ITAL)	2ICA	138	8487
Tyrosine-protein kinase LCK (LCK)	2OF2	420	27374
Trypsin I (TRY)	2AYW	449	25914
Poly [ADP-ribose] polymerase-1 (PARP1)	3L3M	508	3035
Human Immunodeficiency Virus type 1 Protease (HIV)	1XL2	536	35688
Acetylcholinesterase (ACE)	1E66	453	26233
Stem cell growth factor receptor (KIT)	3G0E	166	10447

### Molecular dynamics

The starting structures for the molecular dynamics simulations were the PDB receptor-ligand complexes (described above). The inclusion of the crystallographic ligand was done to avoid an over-compactness of the active site. The PROPKA [49] module of PDB2PQR software package [50, 51] was used to determine the protonation state of the ionizable side chains at pH 7.0. The complexes were protonated using the GROMACS tools [52, 53]. The final model was solvated with a water box ensuring that all protein–ligand atoms are at least 10 Å separated from the edges of the box, using the transferable intermolecular 3 point water model (TIP3P) [54]. The solvated systems were neutralized using Cl^−^ and Na^+^ ions. The AMBER99SB-ILDN force field [55] was used to model the protein. Antechamber, from AMBER tools, was used to calculate the General Amber Force Field (GAFF) [56] parameters for the ligands. All the systems were minimized until the maximum force was less than 1000 kJ/mol.nm using the steep descent algorithm.

MD simulations were carried out using periodic boundary conditions. A spherical cutoff of 1.2 nm for the non-bonded interactions was applied together with a switch function acting between 1.0 and 1.2 nm. The non-bonded list was updated every 20 steps. The particle mesh Ewalds method was used to compute long-range electrostatic term and the leapfrog algorithm was used to propagate the equations of motion. All bond angles involving hydrogen atoms were constrained using the LINCS algorithms [57]. The equilibration, of the solvated protein–ligand system, was carried out in two steps. The first step consisted of 100 ps of the NVT (constant number of particles, volume and temperature) simulation at 300.15 K, in which the protein and ligand heavy atoms were restrained, using a force constant of 1000 kJ/mol.nm. The second step consisted of 100 ps of NPT (constant number of particles, pressure and temperature) simulation at 300.15 K, in which the protein and ligand heavy atoms were restrained, using a force constant of 1000 kJ/mol.nm. MD productions were then carried out without restrains. The time step was set to 2 fs.

To assess the Flexi-pharma method, we used 100, 50, 20 or 5 frames obtained by periodic selection from MD simulations using 10, 5 or 1 ns. The ligand was removed from the MD frames to obtain the ligand-free receptor conformations, which were used to build the pharmacophores (as described above).

### Alternative pharmacophore-based programs

#### Pharmit

We used Pharmit [58] to generate pharmacophoric features from the two crystallographic structures used for each training system. From the set of features (generated by default), all combinations of 4 features were used to generate a set of pharmacophores. We performed a virtual screening with the list pharmacophores using the Pharmer program [34], obtaining a list of potential ligands.

#### Pharmagist

Pharmagist is a ligand-based program that requires known ligands as templates. We used the default input parameters. For the sake of comparison, given that Flexi-pharma does not use training ligands to build the pharmacophores, we implemented pharmagist using just three ligands. We defined two ligand-training sets. For the first set (set 1), we randomly selected three ligands that were not included in the compound library (Table S2). For the second set of template-ligands (set 2), we used the two ligands from the two crystallographic structures for each training system, and a ligand chosen randomly from the compound library. For set 2, the third ligand was chosen randomly five times (Table S3). The ligand with less rotatable bonds was use as a pivot, which is the reference to generate all the pharmacophores. The Pharmagist score was use to calculated the EPs and the EFs.

### Metric validation

To assess the performance of the protocol, two metrics were used: the Enrichment Factor (EF) and the Enrichment Plot (EP).

The EFx% is the ratio between ligands (Hits) found in a certain threshold (x%) of the best ranked compounds and the number of compounds at that threshold (Nx%) normalized by the ratio between the hits contained in the entire dataset (Hits100%) and the total number of compounds N100%.

$$\begin{aligned} EF_x=\frac{Hits^{x\%}}{N^{x\%}}\times \frac{N^{100\%}}{Hits^{100\%}} \end{aligned}$$

Values of EFx% higher than 1, indicate an enrichment of the compound library.

The EPs assess the performance of a filtering method at different levels of the sorted compound library. The EP is a plot of the percentage of ligands found against the percentage of molecules screened [59].

To assess the error of the EPs obtained with Flexi-pharma a bootstrapping analysis with replacement was used. For each MD trajectory the selected frames were iteratively re-sampled with replacement 100 times. Thus 100 EPs were obtained for each trajectory. From these the average and the standard deviation of the EPs were calculated. We note that the bootstrapping technique measures the uncertainty of the results due to the sample size. However, other sources of error (e.g. the grid-energy definition and threshold, number of features, etc.) are not included in this estimate.

## Results and discussion

We present a protocol for virtual screening based on pharmacophores, Flexi-pharma, which uses multiple free ligand–receptor conformations. A possible advantage of our protocol, over structure-based and ligand-based pharmacophore protocols, is that it does not require ligand–receptor complexes or known active compounds. Therefore, the explored pharmacophoric space is not biased by the chemical nature of the known ligands. Additionally, this protocol includes the flexibility of the receptor, which improves the results from virtual screening, as we will show in this .

### Protocol training and optimization

The Flexi-pharma protocol was applied over four benchmark systems used as training set, the cyclin dependent kinase 2 (CDK2), estrogen receptor α (ER), cyclooxygenase-1 (COX) and Glycinamide ribonucleotide transformylase (GAR) (see “Methods” section). The training set was used for studying the behavior of several protocol parameters (e.g. starting structure, grid-energy threshold, number of MD frames, etc.) and for selecting their optimal value.

To assess the dependence of the protocol on the starting structure, two crystal structures with conformational differences in the active site were chosen for each benchmark system of the training set. A compound library was selected for each system. The library consisted of a set of ligands (i.e., active compounds) and decoys (i.e., non-active compounds). We used two metrics to assess the performance of the Flexi-pharma protocol: the Enrichment Factor (EF) and the Enrichment Plot (EP) (see “Methods” section). These metrics measure the enrichment of a compound library after virtual screening. Finally, we compare the performance of our protocol to other state-of-the-art pharmacophore-filtering methods.

#### Flexi-pharma improves outcome in comparison to a single crystal structure

**Fig. 3:**
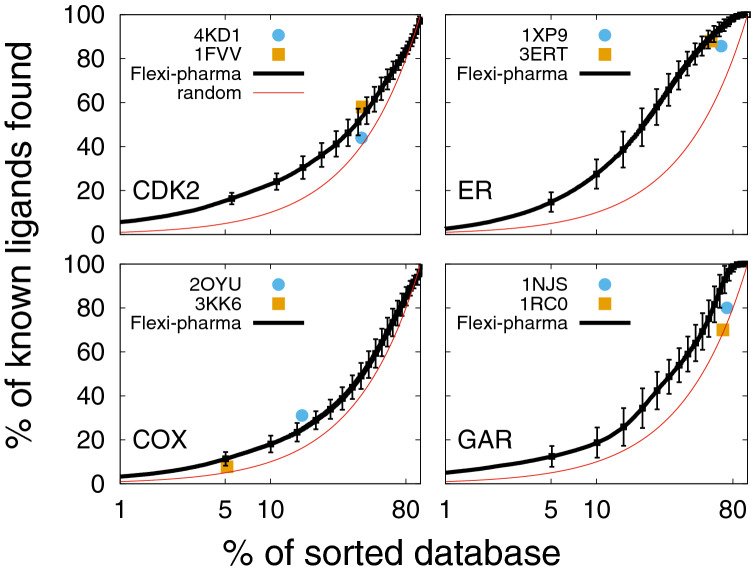
Average enrichment plot obtained after applying Flexi-pharma over four benchmark systems (black curves). For each benchmark system, two starting structures (4KD1 and 1FVV for CDK2; 1XP9 and 3ERT for ER; 2OYU and 3KK6 for COX and 1NJS and 1RC0 for GAR) were used. The MD simulations were 10 ns long, and for each starting conformation they were triplicated by assigning random initial velocities (i.e., 6 MD trajectories for each system). From each trajectory 100 equidistant frames were selected, and the Flexi-pharma protocol was applied. The list of votes is used to calculate the EPs. Bootstrapping analysis was performed by sampling with replacement 100 times to obtain the average EP and standard deviation. The Flexi-pharma protocol was applied using a grid-percentage threshold value of 0.7%. The point corresponds to the % of molecules filtered versus the % of ligands found by applying the protocol only using the crystallographic structure. We note that there are two crystal structures for each system. The x-axis is in logarithmic scale

The application of Flexi-pharma, using multiple receptor conformations from MD, has several advantages over the use of a single crystallographic structure. In Fig. 3, we compare the EPs from the Flexi-pharma protocol for the benchmark systems to the results for only the crystal structures. In an EP the rank of each molecule is used to calculate the percentage of true ligands found as a function of the % of top sorted compounds. We note that the use of a single conformation (e.g., the crystal structure) has the limitation of not supplying a score for the compounds but rather a binary list of possible active compounds, which corresponds to a single point on the EP (Fig. 3). This is a disadvantage because there is no control over what percentage of the library is filtered / selected in the binary list. For example, the list of potential ligands, could be too large for testing experimentally (e.g., EP for GAR in Fig. 3 (blue and orange points) and EFs in Tables S4-S7). In contrast, the use of the Flexi-pharma voting strategy results in a ranking of the molecules, which allows us to select any fraction of the best ranked compounds (e.g., 5% of the compound library) for further screening.

Figure 3, shows that the Flexi-pharma protocol produces better or equal EPs (or EFs) in comparison to using a single crystallographic structure. It is also worth noting that overall the EFs obtained in the range 5–10% of the best ranked compounds are larger than 1, resulting in an enrichment of the database. These results show that the use of Flexi-pharma, with multiple receptor conformations, is suitable for virtual screening of large compound libraries. We highlight that this protocol is not limited to the conformations from MD but can be used with different free ligand–receptor structures from X-ray crystallography, NMR experiments or conformations generated from homology modelling or other simulation techniques.

The EPs showed in Fig. 3 involved several parameters, such as the grid-percentage threshold value, the MD starting structure, the length and number of frames. In the following, we study the influence of these parameters over the results.

#### Grid-percentage threshold independence

**Fig. 4:**
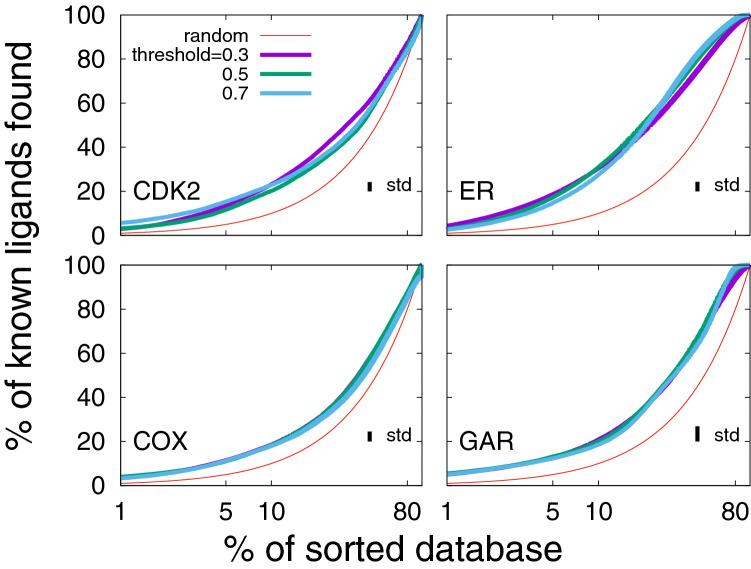
Average enrichment plots obtained after applying the Flexi-pharma protocol over the benchmark systems using different grid-percentage threshold values: 0.3, 0.5 and 0.7%. For each benchmark system, two starting structures (4KD1 and 1FVV for CDK2; 1XP9 and 3ERT for ER; 2OYU and 3KK6 for COX and 1NJS and 1RC0 for GAR) were used. The MD simulations were 10 ns long, and for each starting conformation they were triplicated by assigning random initial velocities (i.e., 6 MD trajectories for each system). From each trajectory 100 equidistant frames were selected, and the Flexi-pharma protocol was applied. The list of votes is used to calculate the EPs. Bootstrapping analysis was performed by sampling with replacement 100 times to obtain the average EP and standard deviation. See the Methods for details about the statistical analysis. The standard deviation (std) of the average enrichment (obtained by bootstrapping analysis) is shown in the inset of each plot. The x-axis is in logarithmic scale

The protocol proposed in this work involves several parameters for defining the set of pharmacophores, among these is the grid-percentage threshold (see the “Methods”). It is used to define the hotspots for the pharmacophoric features in the binding site. In Fig. 4, we show the average EPs for the each system for grid-percentage thresholds of 0.3, 0.5, and 0.7%. We find that the Flexi-pharma results are almost independent for a wide range of grid thresholds. In other words, the EPs calculated at different threshold values do not show statistical differences between each other. However, we note that for a single structure, the number of filtered compounds depends on the threshold value (e.g., see Supplementary Tables S4-S7 for the crystal structures). A large threshold implies a large number of features, which increases the pharmacophore set and the computational time to carry out the virtual screening. Therefore, a good computational efficiency is obtained with small threshold values, while maintaining the performance. Henceforth, the results are shown for the threshold value of 0.7%. However, smaller values can also be used.

#### Outcome for different MD setups

**Fig. 5:**
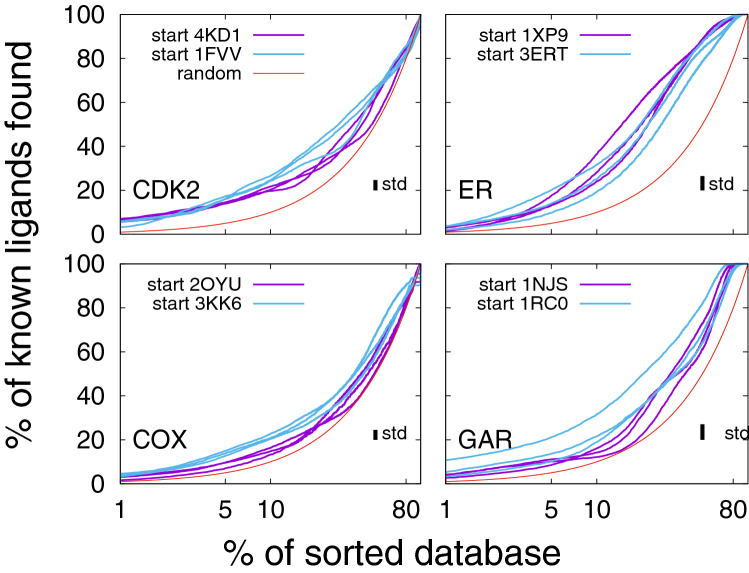
Enrichment plots after applying Flexi-pharma over 6 MD trajectories starting from two crystal structures for each system (4KD1 and 1FVV for CDK2; 1XP9 and 3ERT for ER; 2OYU and 3KK6 for COX; 1NJS and 1RC0 for GAR). Each simulation was 10 ns long, and 100 equidistant frames were selected to apply the Flexi-pharma protocol. Bootstrapping was use to calculate the average EPs for each trajectory. The Flexi-pharma protocol was applied using a grid-percentage threshold value of 0.7%. The standard deviation (std) of the average enrichment (obtained by bootstrapping analysis) is shown in the inset of each plot. The x-axis is in logarithmic scale

We study how the outcome of Flexi-pharma depends on the starting MD structure, number of replicas, number of MD frames and MD simulation length. We used two starting structures for each training system and ran three MD replicas for each starting structure. In Fig. 5, we show the EPs for each replica and each starting structure is labeled with a different color (blue or purple). We find that in almost all cases the results are within statistical error. However, for GAR there seem to be differences between the starting structures, with 1RC0 being slightly better. To investigate this further, we ran two additional simulations starting from each crystal. In Fig. S1, we compare these results (dashed lines) to the ones shown in Fig. 5 (solid lines) for GAR. Interestingly, for the additional simulations, starting crystal 1NJS has the highest enrichment. These results indicate that the starting structure does not significantly change the outcome but several replicas should be performed to have a good convergence. It is worth highlighting the important role of the MD search, which helps decrease the possible dependence of the EPs on the starting structure. In contrast, there is a large dependence observed in the EFs obtained for the crystallographic structures (Tables S4-S7, first row of Table 2 or from docking studies [13, 60, 61]).

**Fig. 6:**
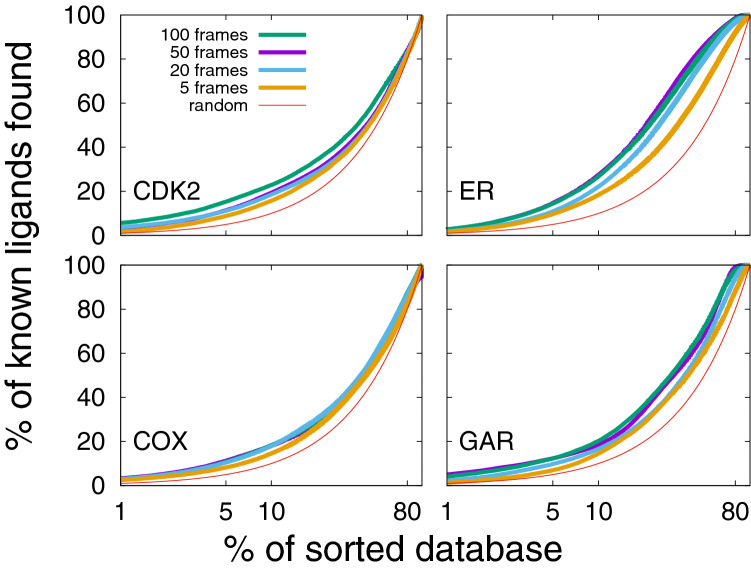
Average enrichment plot obtained after applying the Flexi-pharma over the benchmark systems, using a different number of frames from the MD trajectories. 100, 50, 20 or 5 equidistant frames were selected. Bootstrapping analysis was used to calculate the average EPs for each number of selected conformations. Each trajectory was 10ns long and the grid-percentage threshold was 0.7%. The x-axis is in logarithmic scale

We assess the performance of the Flexi-pharma protocol depending on the number of frames selected from the MD trajectory. In Fig. 6, we show the average EPs for each system and different number of frames at a fixed MD length of 10ns. We find that the larger the number of frames, the slightly better the enrichment obtained. 100 frames (green line Fig. 6) ensures good EPs, however, using 50 frames (purple line Fig. 6) supplies us with similar results at lower computational resources. In contrast, a small number of frames (e.g., 5) results in worse EPs, and a larger error (given that there is a higher probability to obtain the same number of votes among molecules). Thus, a sufficiently large number of conformations is necessary to obtain a good sampling of the binding site and to provide statistical robustness.

**Fig. 7:**
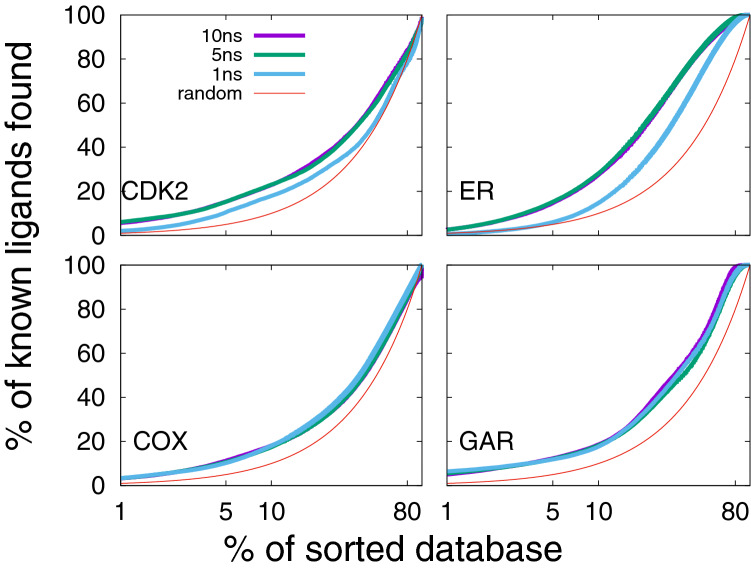
Average enrichment plot obtained after applying Flexi-pharma over the four benchmark systems using MD simulations of different length: 10, 5 and 1 ns. For each individual MD trajectory, 100 equidistant frames were selected. Bootstrapping was used to calculate average EPs for 6 replicas starting from two crystal structures of each system. The Flexi-pharma protocol was applied using a grid-percentage threshold value of 0.7%. The x-axis is in logarithmic scale

A reason for using MD in the Flexi-pharma protocol is that, if significant sampling of the binding site is performed, then the pharmacophores obtained should cover sufficiently well the pharmacophoric space. To assess the performance of Flexi-pharma with the MD length, we calculated the EPs using 100 equidistant frames coming from MD trajectories of 1, 5 and 10 ns. The results are shown in Fig. 7. We found that 10 or 5 ns give similar results. Thus, relatively short MD simulations supply enough conformational sampling of the binding site, decreasing the computational cost of the protocol. However, in some cases too short simulations (CDK2 and ER for 1 ns) are insufficient.

Overall, our results indicate that Flexi-pharma does not have large dependencies on the MD trajectory starting conformation, length or number of selected frames. For example, good enrichment for all the systems in the training set are obtained at the top 5% of the compound library (see Fig. 3 and Table 2 top-row).

**Table 2:** EF5% obtained with Flexi-pharma and Pharmagist for the benchmark systems in the training set. To obtain the EFs with Flexi-pharma, 6 MD trajectories of 10ns (starting from two crystal structures), 100 frames per trajectory, and a grid-percentage threshold of 0.7% were used. For Pharmagist [62, 63] we used two sets of training ligands. The first set uses three ligands that are not included in the compound library as templates. The second uses ligands that are contained in the compound libraries (see “Methods” section)

	CDK2	ER	COX	GAR
Flexi-pharma	3.1	2.9	2.3	2.6
Pharmagist set 1	1.6	0.2	1.4	1.2
Pharmagist set 2	2.6	3.4	3.0	19.3

#### Comparison to other pharmacophore-based methods

In this section, we performed a preliminary comparison of the Flexi-pharma performance to that of a structure-based pharmacophore method: Pharmit [58], and of a ligand-based pharmacophore method: Pharmagist [62, 63] using the training set systems. Pharmit [58] generates pharmacophoric features, from ligand–receptor structures, based on the interactions present in the complexes. We used the two crystal structures for each system as the reference to create the pharmacophores from Pharmit (see “Methods” section for details). In Fig. S2, we compare the results from Flexi-pharma to the individual EFs obtained from each crystal structure. We find that our protocol in most cases has higher EFs than Pharmit. Similarly as with single conformations, Pharmit has the limitation of generating a binary output for each ligand–receptor complex, impeding the ranking of molecules and the selection of different filtering ranges.

**Fig. 8:**
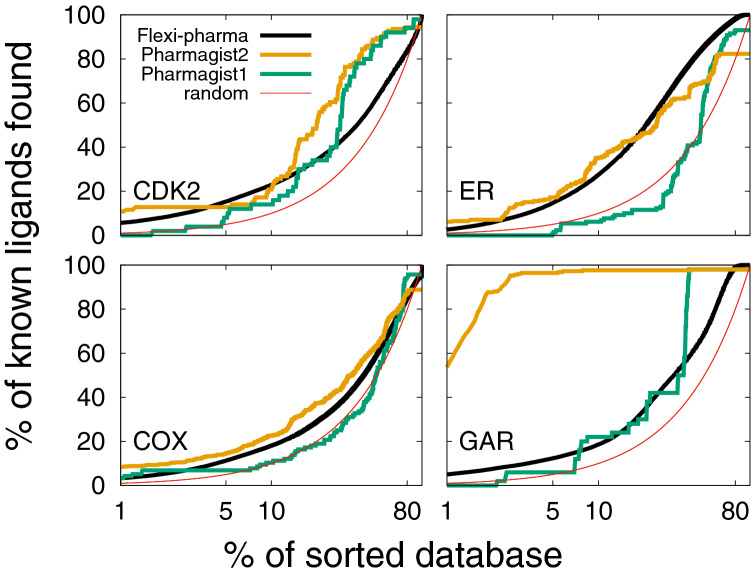
Comparison between EPs obtained with Flexi-pharma and Pharmagist [62, 63]. The average enrichment plot obtained after the application of the Flexi-pharma to four benchmark systems (using 10ns, 100 frames of MD and threshold=0.7%) is showed as a black line. The green line is the EP obtained with Pharmagist using as templates three ligands which were not included in the compound library (set 1). The orange line corresponds to the EP obtained with Pharmagist (set 2), when the pharmacophores were generated using the two ligands from the two crystallographic structures (for each system) and one ligand chosen randomly from the compound library. The third ligand was chosen randomly 5 times to obtain an average EP for Pharmagist set 2

Pharmagist [62, 63] is a ligand-based method that creates consensus pharmacophores from the alignment of know ligands. To carry out a virtual screening with this program, we used two sets of ligands. The first set consists of three known ligands that are not included in the compound library (see “Methods” section). For the second pharmagist training set, we use the two ligands from the two crystallographic structures, and a ligand chosen randomly from the compound library of each system. We note that the second set contains training ligands very similar to those from the compound libraries. From the Pharmagist score, we can calculate the EP and EFs. In Table 2, we compare the EF5% of Pharmagist to the Flexi-pharma outcome for the two sets of training ligands. Also, in Fig. 8, we show the corresponding EPs. An important result that we find is that the Pharmagist outcome is highly dependent on the training set. For the first training-ligand set (those that are not extracted from the compound library; green line in Fig. 8), the Pharmagist results are worse than those obtained with Flexi-pharma in the range 1-40% of the screened dataset. However, for the second set, the Pharmagist results improve significantly. Thus, the results obtained with Pharmagist have large dependence on the ligand templates. On the other hand, Flexi-Pharma does not present this bias, indicating that Flexi-pharma is not limited by the knowledge of ligands or ligand–receptor structures.

### Flexi-pharma over the test set results in good enrichment

We tested the Flexi-pharma protocol over sixteen additional systems (test set described in the Methods). We used one starting crystal structure, the 0.7%, 0.5% and 0.3% grid-energy thresholds, three replicas of 10ns MD simulations, and 100 equidistant MD frames per replica. The EFs at 5 and 10 % are shown in Table 3 for the 0.7% grid-energy threshold together with the EFs from the training set (last four rows). Interestingly, we find that 19 out of the 20 systems show an enrichment of the dataset, with average EFs of 2.8 and 2.3 at 5 and 10 %, respectively. In Figs. S3 and S4, the EPs for the test systems are presented. Remarkably, some of the test systems perform better than those from the training set. These results demonstrate that the Flexi-pharma protocol allows us to discriminate ligands from inactive compounds for a variety of systems. We note that the PA2GA system does not present an enrichment of the dataset. A possible explanation for its failure is that there is a calcium ion in the binding site making direct contact with the ligand. Heavy atoms are not commonly used for the parameterization of the grid energies, which could lead to incorrect features for the pharmacophores. Moreover, for certain force-fields, heavy atoms can destabilize the conformations in the MD simulations. Therefore, the Flexi-pharma protocol might not work well for receptors with heavy atoms in their binding site.

**Table 3:** EF5% and EF10% obtained with Flexi-pharma over the test set using a threshold value of 0.7%, 3 MD replicas of 10 ns long, 100 equidistant frames and bootstrapping to calculate the average EFs. For reference, in the last four lines, we show the EFs for the systems from the training set

System	EF5	EF10
KITH	6.6	5.0
FABP4	4.5	3.1
PA2GA	0.2	0.5
NRAM	7.6	6.0
FA7	8.2	6.6
HSP90a	2.3	2.2
AMPC	2.1	2.6
FKB1A	3.7	2.2
ITAL	3.0	2.3
HXK4	1.2	1.2
TRY	1.7	1.6
ACE	1.6	1.5
HIV	1.4	1.3
PARP1	1.7	1.5
KIT	2.3	2.0
LCK	3.8	2.9
CDK2	3.1	2.3
ER	2.9	2.8
COX	2.3	1.8
GAR	2.6	1.9

### Computational performance

An advantage of the Flexi-pharma protocol over more sophisticated methods like docking is its capacity to screen large compound libraries in a relatively short time. For example, its implementation takes 2.5 minutes per conformation/MD-frame in an single core (processor: Intel Xeon Bronze 3104 CPU @ 1.70GHz) for screening 7987 molecules using the 3ERT structure with a grid-percentage threshold of 0.7% and three active spaces. In contrast, docking of the same library over the same target structure using the vina software [64], rdock [65] or autodock [35], takes approximately 11980, 39935, 143766 minutes, respectively, over a single core using the same processor as before (also see ref. [13]). Because Flexi-pharma is faster but less accurate than the docking-based strategies, the protocol is suitable to use in the first steps of a virtual screening, previous to a docking strategy. However, if one wants to use multiple conformations from MD, one should also take into account the MD simulation time. For the tested systems, to run 10 ns, the average time was 2.8 hours on two of the same processors and with a GPU NVIDIA Tesla P4 8GB Passive.

## Conclusions

We have developed a protocol, Flexi-pharma, that is able to distinguish between active and inactive compounds towards a specific protein receptor, enriching the outcome for 19 out of 20 benchmark systems. Flexi-pharma obtains pharmacophores without the requirement for known active compounds. It is very appropriate for virtual screening over new protein targets, whose binding sites have not been widely characterized, such as allosteric sites.

Flexi-pharma incorporates the flexibility of the binding site in the virtual screening using multiple receptor conformations, which can be obtained from MD simulations. Nevertheless, the flexibility can be introduced using structures from experimental methods (e.g., X-Ray crystallography or NMR) or from homology modelling. The ranking-by-vote strategy allows one to obtain a score for each molecule of the compound library. This is an advantage for selecting any range of the library for further analysis. In addition, the inclusion of flexibility in the pharmacophore-based virtual screening significantly improves the results compared to the use of a single receptor structure.

The protocol has a good performance based on twenty systems. Moreover, it has a robust performance over a wide parameter range, such as the grid-percentage threshold or MD setup. For example, good EF5% are obtained using short 5ns MD simulations with 50 frames, which allow for high computational efficiency. The results indicate that the protocol is suitable to implement over large compound libraries as a first-screening tool, given its demand of few computational resources.

In comparison to pharmacophore-based scoring strategies that use MD simulations [19, 29], the Flexi-pharma method has several differences. First, it avoids generating representative pharmacophores. By clustering the pharmacophores and reducing them to a small set of representative models some underlying information of the receptor’s dynamics could be lost. Second, Flexi-pharma takes directly into account the conformational ensemble (instead of the pharmacophore ensemble) by assigning a vote per conformation (not per pharmacophore). Third, Flexi-pharma does not build the pharmacophores using knowledge of active ligands, which allows for a broad exploration of the chemical space. However, we note that a thorough and extensive comparison to multiple state-of-the-art pharmacophore-based methods might be required to determine which strategy performs best. This large-scale assessment exceeds the scope of this paper that is to present the Flexi-pharma method and its potential applicability.

## Electronic supplementary material

Below is the link to the electronic supplementary material.

Supplementary material 1 (pdf 1322 KB)
