# Supplementary material for: Flexi-pharma: a molecule-ranking strategy for virtual screening using pharmacophores from ligand-free conformational ensembles
Source: J Comput Aided Mol Des. 2020 Jul 12;34(10):1063–77. doi: 10.1007/s10822-020-00329-7 (PMC7449997; doi:10.1007/s10822-020-00329-7)
Supplement: Supplementary file 1 — Supplementary material 1 (pdf 1322 KB) [file 10822_2020_329_MOESM1_ESM.pdf]

Supplementary Information
Flexi-pharma: a molecule-ranking strategy for virtual screening using pharmacophores from ligand-free conformational ensembles

Isaias Lans[1], Karen Palacio-Rodríguez[1], Claudio N. Cavasotto[2,3,4], and Pilar Cossio[1,5,*]

[1]Biophysics of Tropical Diseases Max Planck Tandem Group, University of Antioquia, Medellín, Colombia
[2]Computational Drug Design and Biomedical Informatics Laboratory, Translational Medicine Research Institute (IIMT), CONICET-Universidad Austral, Pilar, Buenos Aires, Argentina
[3]Facultad de Ciencias Biomédicas, and Facultad de Ingeniería, Universidad Austral, Pilar, Buenos Aires, Argentina
[4]Austral Institute for Applied Artificial Intelligence, Universidad Austral, Pilar, Buenos Aires, Argentina
[5]Department of Theoretical Biophysics, Max Planck Institute of Biophysics, 60438, Frankfurt am Main, Germany
[*]Corresponding author: pilar.cossio@biophys.mpg.de

June 20, 2020

1 Supplementary Tables

Table S1: Centers of the three active spaces used for each benchmark system. The first four are the training set and the following systems correspond to the test set.

System	Centers for the active spaces
CDK2	O-Glu81, N-Leu83 and CG-Leu134
ER	CB-Ala350, OE1-Glu353 and NH2-Arg394
COX	O-Ser353, O-Leu352 and CD2-Leu531
GAR	CD1-Leu85, ND2-Asn106 and CG1-Val139
KITH	CZ-Tyr187, CD1-Leu124 and N-Arg180
FABP4	OH-Tyr128, CZ-Phe16 and NH2-Arg106
PA2GA	ND1His47, CE2-Phe5 and CA-Leu2
NRAM	OH-Tyr409, CD-Arg223 and O-Trp177
FA7	OG-Ser195, OG-Ser19 and CG-His57
HSP90a	CG-Phe138, SD-Met98 and CD1-Leu107
AMPC	NZ-Lys67, O-Ala318 and CE1-Tyr150
FKB1A	CE2-Trp59, OH-Tyr82 and CZ-Phe99
ITAL	CD1-Ile235, CZ-Tyr257 and CG1-Val157
HXK4	CD1-Ile211, CG1-Val62 and CE-Met235
TRY	OG-Ser195, O-Ser214 and S190OG
ACE	O-His440, CZ-Phe330 and CE3-Trp84
HIV	OD1(A)-Asp25, CD1(A)-Ile84 and CD1(B)-Ile84
PARP1	OD1-Asp105, CZ-Tyr246 and CZ-TyrY228
KIT	CD1-Leu799, SG-Cys809 and CG2-Val603
LCK	OD1-Asn369, CD1-Leu371 and CD1-Leu251

Table S2: First set of template-ligands used in Pharmagist [1, 2]. These consists of three ligands not included in the compound library or the used crystal structures. The ligand in the first row was used as pivot.

CDK2	ER	COX	GAR
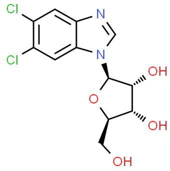<br>ZINC28393967	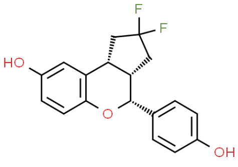<br>ZINC33360187	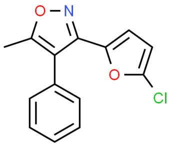<br>ZINC13583263	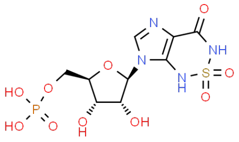<br>ZINC14979160
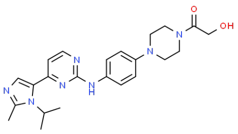<br>ZINC38994180	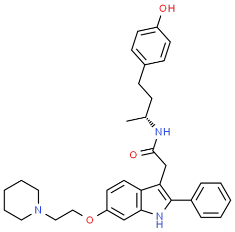<br>ZINC208243905	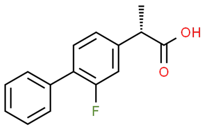<br>ZINC323	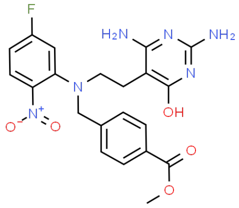<br>ZINC29239617
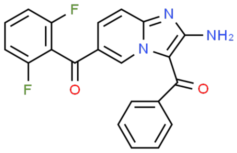<br>ZINC12504093	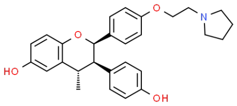<br>ZINC16051697	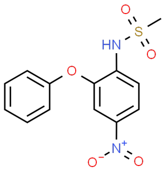<br>ZINC4617749	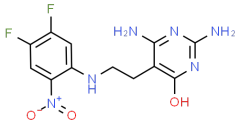<br>ZINC29246271

Table S3: Second set of template-ligands used in Pharmagist [1, 2]. These consists of two ligands from the two crystallographic structures for each system of the training set (first two rows), and a ligand chosen randomly from the compound library. The third ligand was selected randomly 5 times (last five rows). The ligand in the first row was used as pivot.

CDK2	ER	COX	GAR
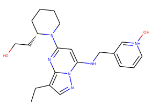 4KD1-ligand	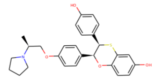 1XP9-ligand	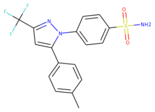 3KK6-ligand	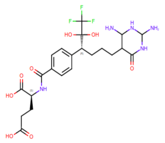 1NJS-ligand
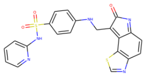 1FVV-ligand	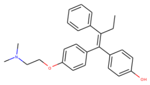 3ERT-ligand	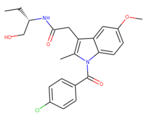 2OYU-ligand	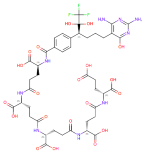 1RC0-ligand
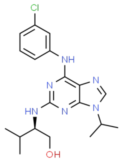 ZINC582575	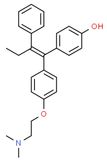 ZINC1530090	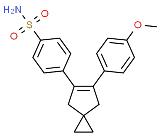 ZINC3814735	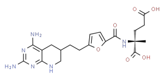 ZINC27880441
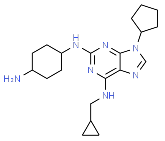 ZINC03814439	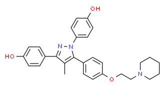 ZINC3996033	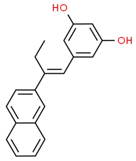 ZINC40891852	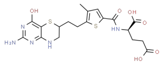 ZINC26505012
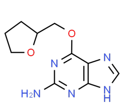 ZINC5988650	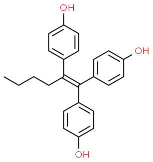 ZINC13494225	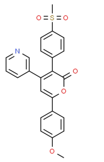 ZINC13581328	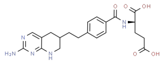 ZINC26580280
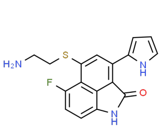 ZINC3814473	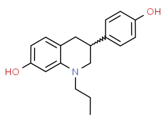 CHEMBL3916194	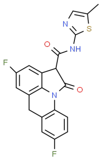 ZINC100658452	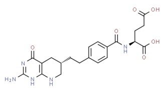 ZINC1637602
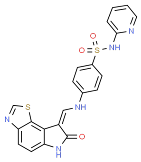 ZINC20149036	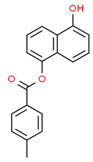 ZINC383800	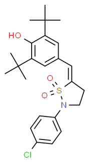 ZINC13558285	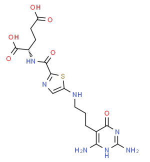 ZINC13737687

Table S4: EFs obtained after implementing the Flexi-pharma over two crystallographic structures of CDK2. The EFs were obtained at different threshold values. Note that the ranking-by-vote strategy is not performed in these cases.

4KD1					1FVV				
Threshold	EF	#ligands	#molecules	EF level	Threshold	EF	#ligands	#molecules	EF level
0.3	1.3	12	346	19%	0.3	1.2	12	372	20%
0.5	1.1	20	670	37%	0.5	1.4	24	606	33%
0.7	1.1	32	739	40%	0.7	1.4	29	747	41%
0.9	1.1	35	1217	67%	0.9	1.1	32	1026	56%
1.1	1.0	37	1320	72%	1.1	1.1	42	1398	76%

Table S5: EFs obtained after implementing the Flexi-pharma over two crystallographic structures of ER. The EFs were obtained at different threshold values. Note that the ranking-by-vote strategy is not performed in these cases.

1XP9					3ERT				
Threshold	EF	#ligands	#molecules	EF level	Threshold	EF	#ligands	#molecules	EF level
0.3	0.4	6	766	12%	0.3	0.6	11	878	13%
0.5	0.3	15	2398	36%	0.5	1.3	114	4345	66%
0.7	1.3	114	4484	67%	0.7	1.5	117	3839	57%
0.9	1.1	125	5853	88%	0.9	1.3	129	4828	72%
1.1	1.1	125	6180	92%	1.1	1.3	132	4952	74%

Table S6: EFs obtained after implementing the Flexi-pharma over two crystallographic structures of COX. The EFs were obtained at different threshold values. Note that the ranking-by-vote strategy is not performed in these cases.

2OYU					3KK6				
Threshold	EF	#ligands	#molecules	EF level	Threshold	EF	#ligands	#molecules	EF level
0.3	2.8	16	353	5%	0.3	0.0	0.0	11	0%
0.5	2.5	34	837	12%	0.5	1.8	9	301	4%
0.7	1.9	36	1153	16%	0.7	1.5	9	365	5%
0.9	2.0	47	1455	20%	0.9	1.7	42	1523	21%
1.1	2.1	66	1894	26%	1.1	1.5	47	1929	27%

Table S7: EFs obtained after implementing the Flexi-pharma over two crystallographic structures of GAR. The EFs were obtained at different threshold values. Note that the ranking-by-vote strategy is not performed in these cases.

1NJS					1RC0				
Threshold	EF	#ligand	#molecules	EF level	Threshold	EF	#ligand	#molecules	EF level
0.3	1.8	37	1134	41%	0.3	18.3	1	3	0%
0.5	1.6	43	1516	55%	0.5	0.8	5	329	12%
0.7	1.1	40	2009	73%	0.7	1.0	35	1882	69%
0.9	1.1	40	2023	74%	0.9	0.9	32	2026	74%
1.1	1.1	49	2371	86%	1.1	1.1	41	2099	77%

2 Supplementary Figures

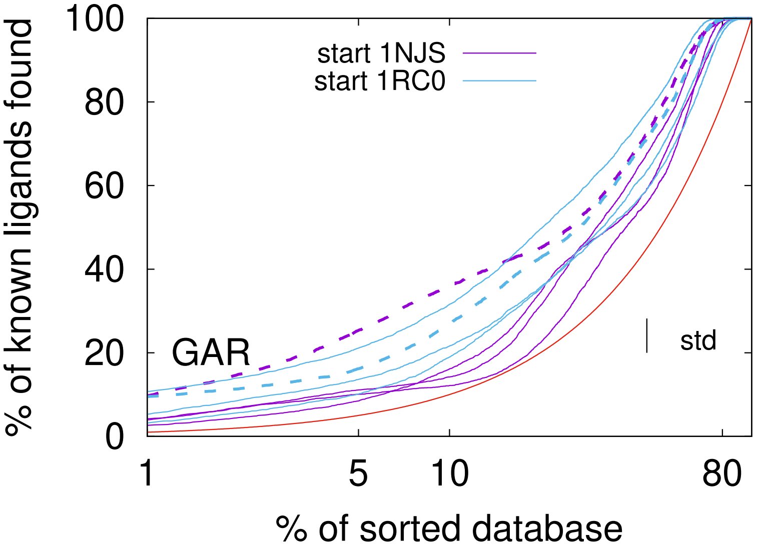

Figure S1: Enrichment plots after applying Flexi-pharma for 2 additional MD replicas starting from the two crystal structures 1NJS and 1RC0 for GAR (dashed lines). For reference, the three initial replicas per starting crystal are shown in solid lines (same data as main text Figure 5 bottom-left). Each simulation was 10 ns long, and 100 equidistant frames were selected to apply the Flexi-pharma protocol. Bootstrapping was use to calculate the average EPs for each trajectory. The Flexi-pharma protocol was applied using a grid-percentage threshold value of 0.7%. The x-axis is in logarithmic scale.

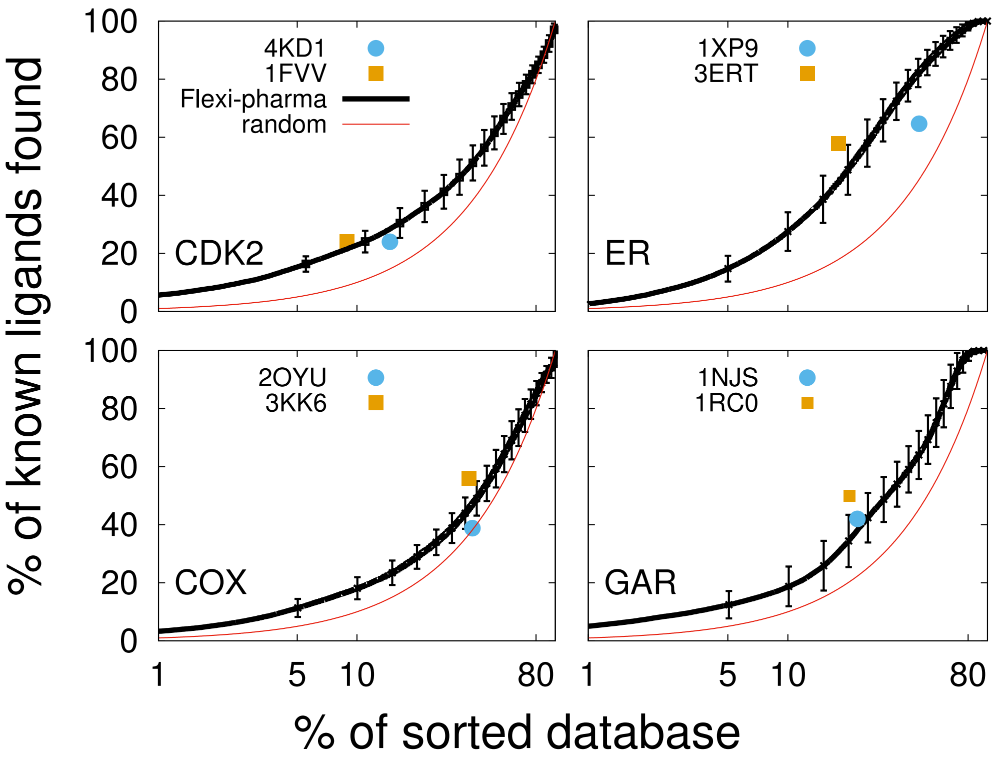

Figure S2: Comparison between the EP obtained with Flexi-pharma (black line) and the results from Pharmit [3]. The point corresponds to the % of molecules filtered versus the % of ligands found by applying Pharmit using each crystallographic structure.

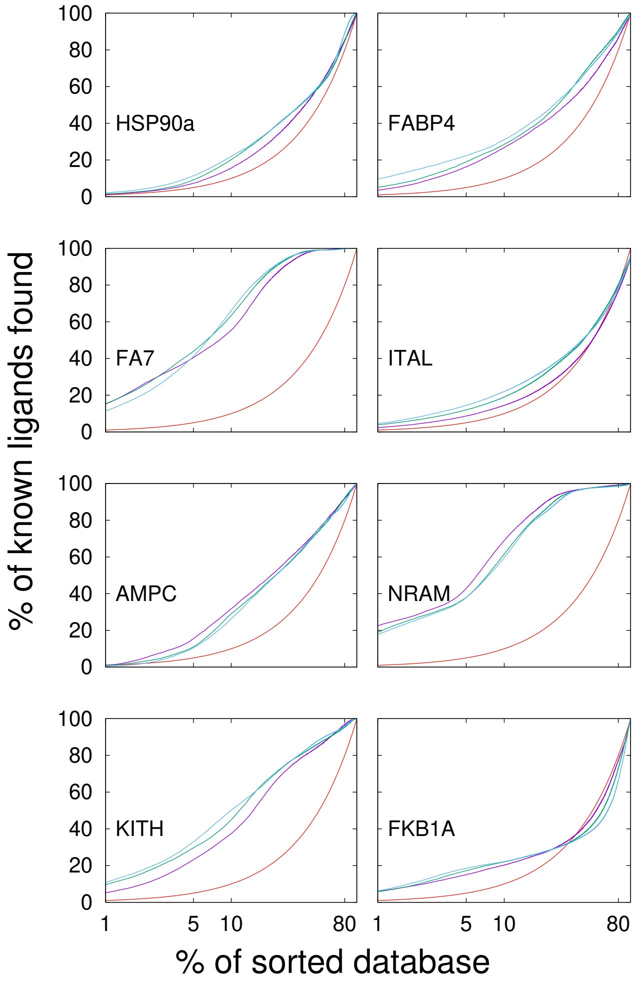

Figure S3: Average enrichment plot obtained after applying Flexi-pharma for 8 test systems: HSP90a (PDB 1UYG), FABP4 (PDB 2NNQ), FA7 (PDB 1W7X), ITAL (PDB 2ICA), AMPC (PDB 1L2S), NRAM (PDB 1B9V), KITH (PDB 2B8T), and FKB1A (PDB 1J4H). The MD simulations were 10 ns long. From each trajectory, 100 equidistant frames were selected, and the Flexi-pharma protocol was applied. The simulations were triplicated by assigning random initial velocities. The list of votes is used to calculate the EPs. Bootstrapping analysis was performed by sampling with replacement 100 times to obtain the average EP and standard deviation. The Flexi-pharma protocol was applied using a grid-percentage threshold value of 0.7% (green), 0.5% (blue) and 0.3% (violet). The x-axis is in logarithmic scale.

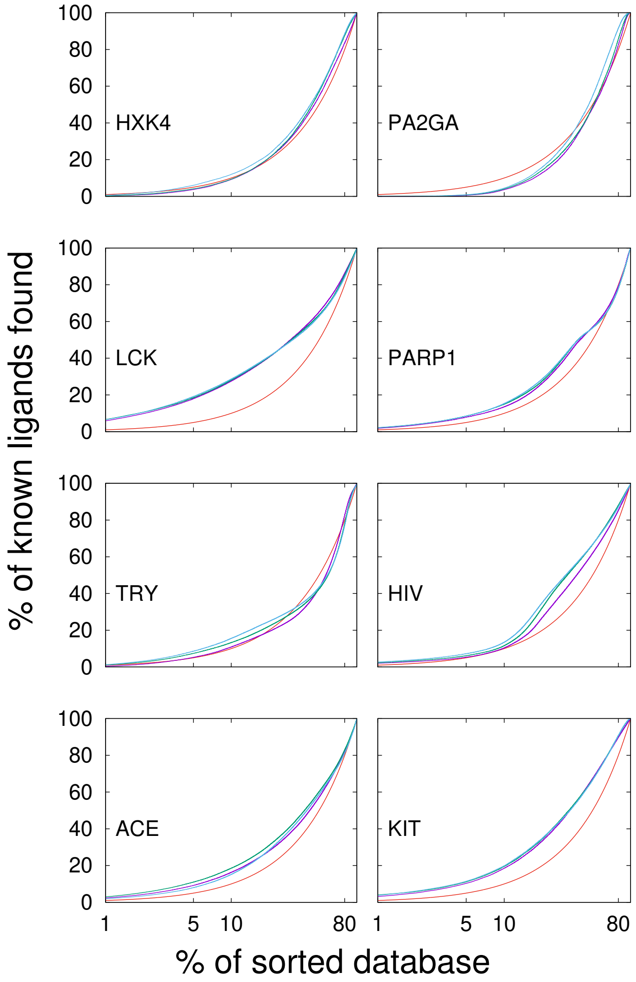

Figure S4: Average enrichment plot obtained after applying Flexi-pharma for 8 systems: HXK4 (PDB 3F9M), PA2GA (PDB 1KVO), LCK (PDB 2OF2), PARP1 (PDB 3L3M), TRY (PDB 2AYW), HIV (PDB 1XL2), ACE (PDB 1E66), and KIT (PDB 3G0E). The MD setup and Flexi-pharma parameters are the same as for Figure S3. The Flexi-pharma protocol was applied using a grid-percentage threshold value of 0.7% (green), 0.5% (blue) and 0.3% (violet). The x-axis is in logarithmic scale.

References

[1] O. Dror, D. Schneidman-Duhovny, Y. Inbar, R. Nussinov, H.J. Wolfson, Journal of Chemical Information and Modeling **49**(10), 2333 (2009)

[2] D. Schneidman-Duhovny, O. Dror, Y. Inbar, R. Nussinov, H.J. Wolfson, Nucleic Acids Research **36**(Web Server), W223 (2008)

[3] J. Sunseri, D.R. Koes, Nucleic acids research **44**(W1), W442 (2016)
